# β-Cell function is associated with osteosarcopenia in middle-aged and older nonobese patients with type 2 diabetes: A cross-sectional study

**DOI:** 10.1515/med-2021-0376

**Published:** 2021-10-20

**Authors:** Jidong Liu, Dongqing Yu, Mingyue Xu, Ruiying Feng, Yujing Sun, Xiaofei Yin, Hong Lai, Chuan Wang, Jinbo Liu

**Affiliations:** Department of Endocrinology, Qilu Hospital, Cheeloo College of Medicine, Shandong University, Jinan, 250012, China; Institute of Endocrine and Metabolic Diseases of Shandong University, Jinan, 250012, China; Key Laboratory of Endocrine and Metabolic Diseases, Shandong Province medicine & health, Jinan, 250012, China; Jinan Clinical Research Center for Endocrine and Metabolic Diseases, Jinan, 250012, China; Department III of Critical Care Medicine, Shandong Provincial Hospital Affiliated to Shandong First Medical University, Jinan, 250021, China

**Keywords:** β-cell function, osteosarcopenia, T2DM, skeletal muscle mass index

## Abstract

Type 2 diabetes mellitus (T2DM) is a strong risk tfactor for osteosarcopenia. The relationship between musculoskeletal index and β-cell function remains controversial. We aimed to describe the clinical characteristics of osteosarcopenia and to explore the association between osteosarcopenia and β-cell function, as well as insulin resistance in patients with T2DM. A total of 150 middle-aged and older nonobese patients with T2DM were recruited. Bone mineral density (BMD) and body composition were measured by the dual-energy X-ray absorptiometry scanner. The homeostasis model assessment of insulin resistance and Matsuda index were used to evaluate insulin resistance status. β-Cell function was estimated by the area under the curve insulin/glucose (AUC-Ins/Glu) and the area under the curve C-peptide/glucose (AUC-CP/Glu). T2DM patients with osteosarcopenia had lower body mass index, waist circumference, body fat percentage, AUC-Ins/Glu, and AUC-CP/Glu. Both AUC-Ins/Glu (OR = 0.634*, P* = 0.008) and AUC-CP/Glu (OR = 0.491, *P* = 0.009) were negatively associated with the presence of osteosarcopenia. Multivariate linear regression analysis showed that β-cell function was positively associated with the skeletal muscle mass index, whereas it showed no correlation with lumbar or hip BMD. β-Cell function is associated with osteosarcopenia in middle-aged and older nonobese patients with T2DM. These findings suggest that β-cell function might be a protective factor against osteosarcopenia.

## 1 Introduction

The term “osteosarcopenia” designates, specifically, the simultaneous presence of sarcopenia and osteopenia/osteoporosis [1,2]. This new concept is now known as geriatric syndrome, which associates with an increased risk of falls, fractures, and impaired mobility. It has been reported that the prevalence of osteosarcopenia ranges from 4.7 to 40% in older adults [3]. The patients with osteosarcopenia had a significantly higher risk of falls and fractures than those having either sarcopenia or osteoporosis [4]. Recently, type 2 diabetes mellitus (T2DM) was considered as a strong risk factor for osteosarcopenia [2].

It has been well known that patients with T2DM tend to have an increased risk of muscle loss and a higher rate of fractures [5,6,7]. The co-occurrence of T2DM and osteosarcopenia may aggravate musculoskeletal health and increase the risk of falls and fractures. However, few studies have focused on the clinical characteristics and risk factors of osteosarcopenia in T2DM patients.

Both insulin resistance and β-cell dysfunction are believed to play a critical role in the pathogenesis of T2DM and its complications [8,9,10]. However, the relationship between musculoskeletal index and β-cell function, as well as insulin resistance, is debated [11,12,13]. Fasting insulin and glucose were used to assess insulin resistance and β-cell function in most of the recent studies, which may not be accurate [14,15]. Euglycemic hyperinsulinemic clamp and hyperglycemic clamp are considered as “gold standard,” but they are costly and not feasible to be carried out in large trials. The concentration of stimulated glucose, insulin, and C-peptide has been recognized as a reliable measure of residual β-cell function and insulin resistance [16,17].

Therefore, this study aimed to investigate the clinical characteristics of osteosarcopenia and to explore the association between the occurrence of osteosarcopenia and β-cell function, as well as insulin resistance in patients with T2DM by using the 75 g-oral glucose test (OGTT).

## 2 Methods

### 2.1 Subjects

A total of 150 (80 men and 70 postmenopausal women) patients with T2DM aged ≥50 years at Qilu Hospital of Shandong University from January 2017 to December 2019. Diabetes was diagnosed based on the 2006 World Health Organization criteria [18]. Patients with a body mass index (BMI) ≤18.5 and ≥30 kg/m^2^ were excluded. Patients with severe liver disease (liver cirrhosis or apparently abnormal liver function defined as serum aspartate aminotransferase or alanine aminotransferase levels >120 U/L), severe kidney disease (estimated glomerular filtration rate [eGFR] <60 mL/min/1.73 m^2^), and any malignant disease or hematologic disease were excluded. This study was approved by the ethics committee of Qilu Hospital of Shandong University.

### 2.2 Clinical evaluation

Age, gender, height, weight, waist circumference, blood pressure (BP), history of smoking and drinking, and medication history of metformin, sulfonylurea, and insulin treatment were assessed on admission. BMI was calculated as weight (kg) divided by height squared (m^2^). Fasting blood was drawn for the measurement of HbA1c, albumin, LDL-C, HDL-C, total cholesterol, triglyceride, and creatinine. At the same time, all diabetic patients underwent 75 g-OGTT. Serum insulin, C-peptide, and glucose concentrations were obtained at 0, 30, 60, 120, and 180 min after OGTT. Insulin resistance was estimated by the homeostasis model assessment of insulin resistance (HOMA-IR) and Matsuda index [19,20]. β-Cell function was measured by the area under the curve insulin/glucose (AUC-Ins/Glu) and the area under the curve C-peptide/glucose (AUC-CP/Glu), which were calculated using the trapezoidal rule applied to the insulin, C-peptide, and glucose curves [20,21].

Lumbar spine and hip bone mineral density (BMD), appendicular lean mass (ALM), and body fat percentage were measured by the dual-energy X-ray absorptiometry scanner (Hologic Inc., Bedford, MA, USA). Lumbar spine or hip BMD based on *T*-score was classified as follows: osteoporosis (*T*-score less than −2.5 SD), osteopenia (*T*-score between −2.5 SD and <(−1) SD), and normal (*T*-score greater than −1 SD). Skeletal muscle mass index (SMI) was calculated as follows: SMI (kg/m^2^) = ALM (kg)/height^2^ (m^2^). Sarcopenia was defined as SMI below a cutoff of 7.0 kg/m^2^ (men) and 5.4 kg/m^2^ (women) [22]. Osteosarcopenia was defined as the presence of osteopenia/osteoporosis and sarcopenia [2]. The eGFR was calculated using the Chronic Kidney Disease Epidemiology Collaboration equation [23].

### 2.3 Statistical analysis

The continuous variables are expressed as the mean ± standard deviation (SD) and the mean (interquartile range). Between-group differences were detected using Student’s *t*-test or the Mann–Whitney *U*-test or chi-square test. The data were graphed using Prism software (Graphpad, California, USA). Binary logistic regression was used to evaluate the risk factors for osteosarcopenia in T2DM patients. The relationship between β-cell function and musculoskeletal indices was assessed by multiple linear regression. The statistical analysis was done using SPSS 22.0 software (SPSS Inc., Chicago, USA), and *P* < 0.05 was considered statistically significant.

## 3 Results

### 3.1 General characteristics of participants

The demographics and laboratory results of all T2DM patients are shown in Table 1. Age and gender distributions were not different between the two groups (nonosteosarcopenia vs osteosarcopenia). The patients with osteosarcopenia showed lower BMI, waist circumference, and body fat percentage. There was no significant difference in the history of smoking and drinking, diabetes duration, BP, lipid profile, renal function, albumin, HbA1c, or antidiabetic medication between the two groups.

**Table 1:** Demographic and clinical parameters of the study population

	Nonosteosarcopenia	Osteosarcopenia	*P*
*n*	106	44	
Age (years)	60.17 ± 6.45	61.84 ± 6.76	0.156
Female	57.5%	43.2%	0.150
Smoking	20.8%	27.3%	0.398
Drinking	23.6%	29.5%	0.537
Diabetes duration months	150 (96–216)	126 (81–204)	0.544
SBP (mmHg)	136.85 ± 19.00	132.66 ± 22.84	0.249
DBP (mmHg)	78.88 ± 10.33	75.14 ± 12.55	0.060
BMI	26.03 ± 2.44	22.75 ± 2.45	**<0.001**
Waist circumference (cm)	95.58 ± 9.51	89.42 ± 9.68	**<0.001**
Body fat percentage (%)	32.37 ± 5.61	29.96 ± 6.37	**0.023**
Total cholesterol (mmol/L)	4.61 (3.90–5.36)	4.36 (3.98–5.14)	0.980
LDL-C (mmol/L)	2.79 (2.32–3.43)	2.67 (2.23–3.13)	0.212
HDL-C (mmol/L)	1.17 (1.06–1.32)	1.23 (1.00–1.54)	0.249
Triglyceride (mmol/L)	1.48 (1.09–2.17)	1.29 (0.91–1.93)	0.649
Albumin (g/L)	43.68 ± 3.92	43.47 ± 4.57	0.790
eGFR (mL/min/1.73 m^2^)	96.67 ± 11.91	98.58 ± 9.59	0.347
HbA1c (%)	8.55 ± 1.87	8.64 ± 1.83	0.720
FBG (mmol/L)	8.12 (5.94–9.47)	8.63 (6.32–10.08)	0.278
0.5 h glucose (mmol)	10.98 (9.09–13.28)	11.52 (9.55–13.28)	0.261
1 h glucose (mmol)	15.28 (13.23–17.88)	16.14 (13.68–18.30)	0.172
2 h glucose (mmol)	18.06 (15.70–20.51)	19.39 (17.30–20.98)	0.061
3 h glucose (mmol)	16.61 (14.04–19.07)	18.04 (16.12–20.06)	0.058
Fasting insulin (mIU/L)	19.32 (8.78–21.95)	16.25 (6.43–21.00)	0.319
0.5 h insulin (mIU/L)	25.76 (14.27–29.97)	19.84 (9.43–25.79)	**0.046**
1 h insulin (mIU/L)	36.55 (21.00–48.49)	27.96 (13.90–35.64)	**0.020**
2 h insulin (mIU/L)	46.20 (23.76–62.63)	36.18 (19.31–43.45)	0.059
3 h insulin (mIU/L)	39.30 (20.89–54.23)	28.01 (15.10–36.53)	**0.010**
Fasting C-peptide (ng/mL)	1.50 (0.76–1.90)	1.20 (0.60–1.55)	0.065
0.5 h C-peptide (ng/mL)	2.00 (1.11–2.49)	1.57 (0.94–2.16)	**0.034**
1 h C-peptide (ng/mL)	2.81 (1.63–3.76)	2.14 (1.24–2.75)	**0.016**
2 h C-peptide (ng/mL)	4.17 (2.42–5.40)	3.52 (2.13–4.33)	0.140
3 h C-peptide (ng/mL)	4.38 (2.71–5.50)	3.40 (1.99–4.44)	**0.018**
AUC-Ins/Glu	2.56 (1.46–3.40)	1.84 (0.93–2.31)	**0.010**
AUC-CP/Glu	0.23 (0.12–0.29)	0.17 (0.09–0.21)	**0.013**
Metformin usage (%)	80.2%	70.5%	0.205
Sulfonylurea usage (%)	51.9%	45.5%	0.591
Insulin usage (%)	52.4%	59.1%	0.476

Data are shown as the mean ± SD, median (interquartile range), or number (%).

SBP, systolic blood pressure; DBP, diastolic blood pressure; BMI, body mass index; LDL-C, low-density lipoprotein cholesterol; HDL-C, high-density lipoprotein cholesterol; and eGFR, estimated glomerular filtration rate.

Significant *P* values (<0.05) are indicated in bold.

### 3.2 β-Cell function is different between osteosarcopenia and nonosteosarcopenia groups

**Figure 1:**
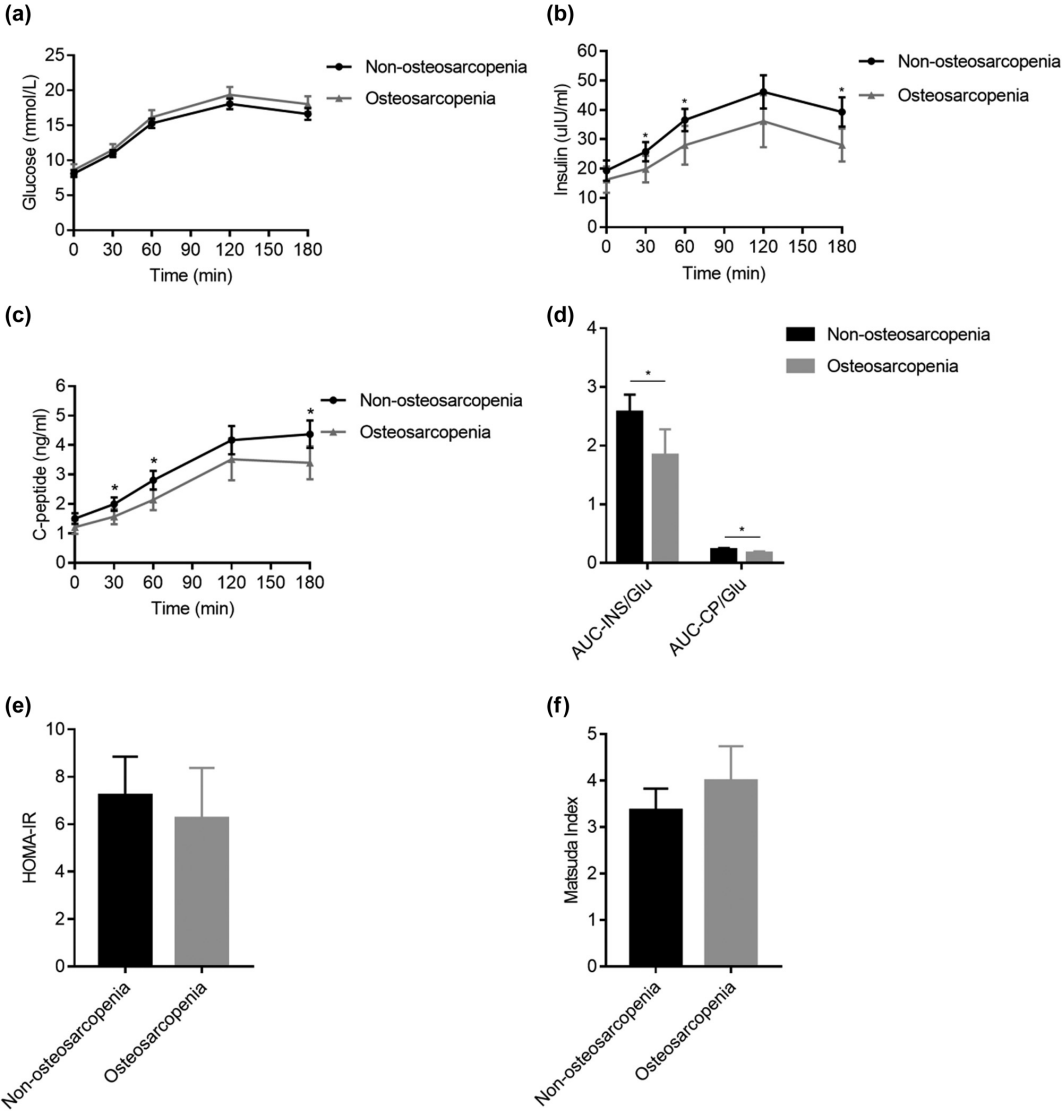
Comparison of the levels of OGTT-induced serum glucose, insulin, C peptide, β-cell function, and insulin resistance status of the two groups. (a–c) Serum glucose (a), insulin (b), and C-peptide (c) during 180 min OGTT. (d) β-Cell function measured by AUC-Ins/Glu and AUC-CP/Glu. (e–f) Insulin resistance status measured by HOMA-IR (e) and Matsuda index (f). Each error bar is constructed using a 95% confidence interval of the mean. *P < 0.05 versus nonosteosarcopenia.

75 g-OGTT was performed to explore insulin resistance and β-cell function in T2DM patients with osteosarcopenia. As shown in Table 1 and Figure 1, fasting serum glucose, insulin, and C-peptide concentrations were not significantly different between the two groups (Figure 1a–c). Postprandial insulin and C-peptide (30, 60, and 180 min) were significantly lower in the osteosarcopenia group compared with nonosteosarcopenia group. Furthermore, patients with osteosarcopenia showed lower AUC-Ins/Glu and AUC-CP/Glu levels (Figure 1d); nevertheless, no difference was observed in HOMA-IR and Matsuda index between the two groups (Figure 1e–f).

### 3.3 β-Cell function is independently associated with the presence of osteosarcopenia in T2DM patients

To elucidate whether β-cell function is independently associated with the presence of osteosarcopenia, binary logistic regression using three models was conducted to assess the participants, as shown in Table 2. Before adjustment, AUC-Ins/Glu and AUC-CP/Glu showed a decreased odds ratio for osteosarcopenia. After adjusting for age, gender, duration of T2DM, medical history of sulfonylurea and insulin, and body fat percentage in model 2, both AUC-Ins/Glu and AUC-CP/Glu were still negatively associated with osteosarcopenia. Smoking and drinking status, renal dysfunction, malnutrition, and hyperglycemia were known causes of osteosarcopenia [2], and after an additional adjustment for these traditional risk factors, β-cell function was still significantly associated with a lower risk of osteosarcopenia. Notably, there was no correlation between the presence of osteosarcopenia and fasting insulin, as well as fasting C-peptide.

**Table 2:** Binary logistic regression analysis of the relationship between β-cell function and osteosarcopenia

	AUC-Ins/Glu		(AUC-CP) ∗ 10/Glu		Fasting insulin		Fasting C-peptide	
Model	OR (95% CI)	*P*	OR (95% CI)	*P*	OR (95% CI)	*P*	OR (95% CI)	*P*
Model 1	0.687 (0.511–0.924)	**0.013**	0.672 (0.485–0.930)	**0.016**	0.988 (0.966–1.012)	0.321	0.655 (0.415–1.033)	0.069
Model 2	0.664 (0.477–0.923)	**0.015**	0.596 (0.392–0.905)	**0.015**	0.987 (0.962–1.012)	0.303	0.603 (0.347–1.046)	0.072
Model 3	0.634 (0.453–0.887)	**0.008**	0.491 (0.287–0.840)	**0.009**	0.980 (0.953–1.008)	0.163	0.745 (0.407–1.364)	0.340

Model 1: not adjusted.

Model 2: adjusted for age, gender, duration of T2DM, insulin and sulfonylurea usage, and body fat percentage.

Model 3: adjusted for age, gender, duration of T2DM, insulin and sulfonylurea usage, body fat percentage, history of smoking and drinking, HbA1C, albumin, and eGFR.

Significant *P* values (<0.05) are indicated in bold.

### 3.4 β-Cell function was positively associated with SMI in T2DM patients

Multiple linear regression analysis was conducted to explore the potential relationship between β-cell function and musculoskeletal indices in T2DM patients. As shown in Table 3, both AUC-Ins/Glu and AUC-CP/Glu were positively associated with SMI, independent of age, gender, history of smoking and drinking, duration of T2DM, medical history of sulfonylurea and insulin, albumin, eGFR, and body fat percentage. No correlation was found between β-cell function and lumbar spine or hip BMD (Table 3).

**Table 3:** Multiple linear regression analysis of β-cell function and musculoskeletal indices in T2DM patients

	AUC-Ins/Glu		AUC-CP/Glu	
	β coefficient (95% CI)	*P*	β coefficient (95% CI)	*P*
SMI	0.084 (0.004–0.164)	**0.040**	0.132 (0.009–0.255)	**0.036**
Lumbar BMD	0.005 (−0.012 to 0.022)	0.591	0.011 (−0.016 to 0.036)	0.446
Hip BMD	0.009 (−0.004 to 0.023)	0.173	0.018 (−0.002 to 0.039)	0.084

Adjusted for age, gender, duration of T2DM, insulin and sulfonylurea usage, body fat percentage, history of smoking and drinking, HbA1C, albumin, and eGFR.

Significant *P* values (<0.05) are indicated in bold.

## 4 Discussion

In this study, we first compared the clinical characteristics of osteosarcopenia and nonosteosarcopenia patients with T2DM. Patients with osteosarcopenia showed lower BMI, waist circumference, body fat percentage, and worse β-cell function. However, neither insulin resistance nor glucose control showed differences between the groups. The presence of osteosarcopenia was found to be negatively correlated with β-cell function independent of other factors, such as age, diabetes duration, history of smoking and drinking, malnutrition, body fat percentage, renal dysfunction, and glucose control.

T2DM patients have lost 50% of β-cell function at the time of diagnosis [24], and this value gradually increases with longer disease duration [25]. Residual insulin secretion capacity is considered a preventive factor against diabetic complications [26]. However, the relationship between musculoskeletal index and β-cell function is controversial. SMI was negatively associated with glucagon-stimulated C-peptide in T2DM patients <65 years, but the correlation was not found between SMI and blood glucose control [11]. In contrast, one study found that endogenous insulin secretion capacity was positively associated with muscle mass, and high HbA1c was associated with the occurrence of sarcopenia in T2DM subjects [12]. Moreover, some studies demonstrated that BMD was positively associated with insulin and C-peptide [27,28], whereas other studies obtained the opposite results [29,30].

Our results differ from the above studies, which may be due to the following several points: First, OGTT-induced glucose, insulin, and C-peptide were measured to reflect β-cell function and insulin resistance status, which are more accurate than fasting insulin and C-peptide [16,17]. Compared with the control group, in our study, no difference was identified in the fasting insulin and C-peptide concentration in the osteosarcopenia group. Neither fasting insulin nor C-peptide was associated with the presence of osteosarcopenia. Second, we studied specifically age-matched and nonobese T2DM subjects with similar disease duration, which can minimize these confounding factors. Third, both insulin secretion capacity and musculoskeletal index are highly correlated with fat mass [31,32,33]. However, few studies have considered the effect of fat mass on the association between β-cell function and musculoskeletal indices. Our study revealed that the association between the presence of osteosarcopenia and β-cell function persisted after adjusting body fat percentage, suggesting that the association is independent of body fat percentage.

Moreover, we found that SMI showed a positive correlation with β-cell function, whereas BMD did not show any correlation. The interaction among pancreas, bone, and muscle is uncertain. Bone and muscle are connected anatomically, and they interact with each other through multiple mechanisms. Several growth factors secreted by myotubes, such as fibroblast growth factor 2 and insulin-like growth factor 1 (IGF1), have an anabolic effect on bone [34], and IGF1 could also promote proliferation and differentiation in osteoblasts [35]. In addition, muscle loss is associated with the deficiency of insulin and C-peptide in type 1 diabetes [36,37]. Insulin exerts an anabolic effect on skeletal muscle either through stimulation of protein synthesis or through inhibition of proteolysis [38,39]. C-peptide has also been found to have a protective effect against cell death in myoblasts [40]. Thus, our findings support the hypothesis that β cells might have an indirect effect on bone through skeletal muscle [41].

To our knowledge, this is the first study to report the correlation of β-cell function with osteosarcopenia in T2DM patients. There were several limitations to our studies. First, this was a cross-sectional study with a small sample size. However, the statistically significant results encourage multiple-center and large-sample studies to verify our findings. Second, we did not measure IGF1 or Vitamin D, which is essential for skeletal muscle maintenance and bone remodeling [35,42]. Finally, sarcopenia was just defined by the SMI, and grip strength or physical performance was not evaluated in this study.

## 5 Conclusion

We found that β-cell function might be a protective factor against osteosarcopenia in middle-aged and older nonobese patients withT2DM. Moreover, this relationship was independent of other osteosarcopenia risk factors, such as age, duration of diabetes, smoking, drinking, malnutrition, and glucose control. These findings suggest that interventions to preserve β-cell function are important for the prevention of osteosarcopenia in patients with T2DM.
